# The Vascular Function of Resistance Arteries Depends on NADPH Oxidase 4 and Is Exacerbated by Perivascular Adipose Tissue

**DOI:** 10.3390/antiox13050503

**Published:** 2024-04-23

**Authors:** Patrick Diaba-Nuhoho, Jennifer Mittag, Coy Brunssen, Henning Morawietz, Heike Brendel

**Affiliations:** Division of Vascular Endothelium and Microcirculation, Department of Medicine III, University Hospital and Faculty of Medicine Carl Gustav Carus, TUD Dresden University of Technology, Fetscherstr. 74, 01307 Dresden, Germany; patrick.diabanuhoho@ukmuenster.de (P.D.-N.); coy.brunssen@ukdd.de (C.B.)

**Keywords:** NADPH oxidase 4, *Nox4*, resistance arteries, endothelial function, perivascular adipose tissue

## Abstract

The NADPH oxidase NOX4 that releases H_2_O_2_ can mediate vasoprotective mechanisms under pathophysiological conditions in conductive arteries. However, the role of NOX4 in resistance arteries and in perivascular adipose tissue is not well understood. We hypothesized that NOX4 is of functional importance in resistance arteries and perivascular adipose tissue under dyslipidemia conditions. We detected elevated *NOX4* expression in murine and human vessels under dyslipidemia. Diminishing *Nox4* under these conditions led to endothelial dysfunction in resistance arteries. The mesenteric arteries of *Nox4*^−/−^/*Ldlr*^−/−^ mice revealed decreased *eNos* mRNA expression. Inhibition of eNOS in those vessels did not affect vascular function, while in *Ldlr*^−/−^ mice endothelial function was significantly altered. Anticontractile properties of perivascular adipose tissue at resistance arteries were diminished in *Nox4*^−/−^/*Ldlr*^−/−^ compared with *Ldlr*^−/−^ mice. In addition, the presence of perivascular adipose tissue further worsened endothelial dysfunction in mesenteric arteries under dyslipidemia conditions. Perivascular adipose tissue from mesenteric arteries revealed a higher expression of markers of white adipocytes compared to markers of beige/brown adipocytes. Among those white adipocyte markers, leptin was significantly less expressed in perivascular adipose tissue from *Nox4*^−/−^/*Ldlr*^−/−^ mice compared with *Ldlr*^−/−^ mice. Furthermore, in human perivascular adipose tissue with a profound pattern of white adipocyte marker genes, we detected a correlation of *NOX4* and *LEP* expression. In addition, incubating arterial vessels with leptin induced nitrite release, indicating increased eNOS activity. In humans, a higher expression of leptin in perivascular adipose tissue correlated with *eNOS* expression in the corresponding left internal mammary artery. In conclusion, vascular function of resistance arteries was dependent on *Nox4*-derived H_2_O_2_, especially under dyslipidemia conditions. Perivascular adipose tissue of the mesenteric arteries with white adipose tissue characteristics further aggravated endothelial function through reduced leptin-eNOS signaling.

## 1. Introduction

Dyslipidemia is one of the major risk factors for cardiovascular diseases and refers to an imbalance in serum levels of low-density lipoprotein cholesterol, high-density lipoprotein cholesterol and triglycerides, contributing to the development of atherosclerosis [1,2]. Dyslipidemia can lead to endothelial dysfunction in resistance arteries, impairing their ability to regulate blood flow and thereby contributing to hypertension and other cardiovascular complications [3]. Resistance arteries are small arteries that play a crucial role in regulating blood pressure by controlling peripheral resistance.

In the presence of dyslipidemia, the function and structure of resistance arteries can be significantly altered. The endothelium as the inner layer of a vessel wall is particularly susceptible to damage from increased levels of circulating and oxidatively modified low-density lipoproteins. This first damage, termed endothelial dysfunction, is characterized by oxidative stress and an imbalance between vasodilator and vasoconstrictor substances [4]. Consequently, endothelial dysfunction leads to increased arterial stiffness, reduced vasodilation, and impaired ability to regulate blood flow, all of which contribute to the progression of cardiovascular diseases [3]. There is evidence suggesting that the onset of endothelial dysfunction in small resistance arteries precedes the development of endothelial dysfunction in conduit arteries [5,6].

Growing evidence indicates that perivascular adipose tissue (PVAT) plays a significant role in vascular homeostasis. PVAT is a unique fat deposit surrounding many blood vessels and influences vascular function through the production and secretion of adipokines that regulate vascular tone and inflammation. Under healthy conditions, PVAT exerts anti-contractile effects that promote vasodilation [7]. PVAT might even contribute to the maintenance of endothelial function in obese patients [8]. However, in states of dyslipidemia, PVAT can become dysfunctional; its beneficial effects are diminished, and it may even contribute to the development and progression of cardiovascular diseases by secreting inflammatory cytokines and adipokines [9]. These factors promote a pro-inflammatory and pro-atherogenic environment, leading to endothelial dysfunction and vascular remodeling [10,11]. The role of the adipokine leptin in this context is not well-understood [12,13].

The NADPH oxidase (NOX) family is the predominant source of reactive oxygen species (ROS) in vessel walls. While ROS play vital roles in cellular signaling and homeostasis, excessive ROS production can lead to oxidative stress, further impairing endothelial function and promoting inflammation [14,15]. Seven NOX isoforms have been described. NOX2 generates superoxide anions and is mainly considered deleterious in the cardiovascular system. In contrast, NOX4 is the major NOX isoform in endothelial cells and mainly an enzymatic source of H_2_O_2_ instead of superoxide anions [16]. Experimental studies support the involvement of NOX4 as a secondary messenger in various biological processes including cell proliferation [17], differentiation [18], ferroptosis [19], oxygen sensing [20] and angiogenesis [21]. Additionally, NOX4 was shown to mediate vasoprotection under pathological conditions such as hypertension [22,23] and atherosclerosis [24]. Thus, NOX4 protects against the development of endothelial dysfunction and atherosclerosis in conduit arteries in LDL-receptor-deficient mice [25]. Loss of Nox4-derived H_2_O_2_ can be partially compensated for by nNOS upregulation, but severe endothelial dysfunction is not reversible. Furthermore, NOX4 mediates the protective effects of physical activity against obesity-induced vascular dysfunction. This involves NOX4-dependent exercise-induced adaptations of eNOS and Ppargc1a pathways and intracellular calcium release.

However, the role of NOX4 in small resistance arteries and in the interplay between perivascular adipose tissue and vasculature remains to be explored.

## 2. Materials and Methods

### 2.1. Mouse Models

All experiments were performed in accordance with the Guide for the Care and Use of Laboratory Animals published by the US National Institutes of Health (NIH Publication No. 85-23, revised 1996). The animal research ethics committee of the Technische Universität Dresden, Germany, and the Regional Council of Saxony (Landesdirektion Sachsen) approved the animal experiments according to institutional guidelines and German animal welfare regulations (AZ: DD24.1-5131/449/21, AZ: DD24.1-5131/449/20).

C57BL/6J/*Ldlr*^−/−^ (*Ldlr*^−/−^) was obtained from The Jackson Laboratory via Charles River (Sulzfeld, Germany). C57BL/6J/*Nox4*^−/−^ (*Nox4*^−/−^) mice were kindly provided by Ralf P. Brandes and Katrin Schröder (Goethe University, Frankfurt, Germany). *Nox4*^−/−^/*Ldlr*^−/−^ mice were generated by crossing *Nox4*^−/−^ mice with *Ldlr*^−/−^ mice in-house for at least five generations and with continuous genotyping.

### 2.2. Human Tissue Samples

Patients were admitted to elective coronary artery bypass grafting surgery at the Heart Center Dresden, Fetscherstraße 76, 01307 Dresden, Germany. All patients received detailed verbal and written information about the study and gave written informed consent. The study was approved by the Ethics Committee of the Technische Universität Dresden (EK 307-12-2007). The investigation conforms with the principles outlined in the Declaration of Helsinki. Clinical characteristics of the patients are listed in Table 1. Dyslipidemia was defined according to the American Heart Association’s classification corresponding to the 95th percentile in an American population as total cholesterol > 5.2 mmol/L (200 mg/dL), LDL > 3.4 mmol/L (130 mg/dL), HDL < 0.9 mmol/L (35 mg/dL), or triglycerides > 1.7 mmol/L (150 mg/dL), or a combination thereof [26].

Left internal mammary artery (LIMA) and perivascular adipose tissue were dissected as described before [8]. Briefly, during coronary artery bypass graft surgery, distal remnant specimens of the left internal mammary artery were obtained from patients. The specimens were dissected approximately 30 min after sternotomy. The tissue samples were immediately stored in 25 mL TiProtec^®^ solution (Köhler Chemie, Bensheim, Germany) at a temperature of 4 °C and transferred to the laboratory. Then the internal mammary artery was separated from perivascular tissue under a microscope and both were quickly shock frozen and stored at −80 °C for further processing.

### 2.3. Murine Tissue Sampling

Weight and food intake were measured before the mice were sacrificed. Animals were sacrificed by cervical dislocation; aorta, mesenteric artery, perivascular tissue from the aorta and perivascular tissue from the mesenteric artery were excised, rinsed with ice-cold PBS and snap-frozen for further analyses.

### 2.4. RNA Isolation and REAL-Time PCR

Tissue was lysed with RNA lysis buffer and homogenized with a Precellys homogenizer (Peqlab Biotechnologie GmbH, Erlangen, Germany). Total RNA was isolated using a peqGold Total RNA Kit (Peqlab Biotechnologie GmbH, Erlangen, Germany) and determined using a NanoDropTM 1000 Spectrophotometer (Peqlab Biotechnologie GmbH, Erlangen, Germany). Reverse transcription was performed with SuperScript II Reverse Transcriptase according to the manufacturer’s instructions (Life Technologies, Darmstadt, Germany). The mRNA expression was determined by SYBR green-based real-time PCR reactions using specific primers. Quantification was performed by real-time PCR with GoTaq qPCR Master Mix (Promega, Mannheim, Germany). Analysis of raw data was performed with 7500 Software version 2.06 (Applied Biosystems by Life Technologies, Darmstadt, Germany). Evaluation of the data was carried out using a mathematical model of relative expression ratio in real-time PCR under constant reference gene expression [27]. The primers used are listed in Table 2.

### 2.5. Vessel Preparation and Vascular Function Analysis

Mesenteric artery segments were isolated from the second and third branches of the mesenteric artery network, cleaned, removed and placed in Krebs-Henseleit buffer (in mM): 173.85 NaCl, 5.9 CaCl_2_.2H_2_O, 8.75 KCl, 7.4 MgSO_4_.7H_2_O, 52.5 NaHCO_3_, 4.1 KH_2_PO_4_ and 4.1 EDTA, with 5% CO_2_ in 95% O_2_ at 37 °C. Vascular function analysis was performed using a DMT Multi Wire Myograph System–Model 620 M (Danish Myo Technology A/S, Hinnerup, Denmark). Endothelial function was tested with acetylcholine (ACh) in rings precontracted with 1 µM phenylephrine (PE). Smooth muscle function was analyzed in precontracted rings with sodium nitroprusside (SNP). In addition, effects of ACh were tested in the presence of catalase (1500 U/mL, 30 min preincubation) or NO synthase inhibitor L-NG-Nitroarginine methyl ester (L-NAME, 300 µM, 30 min preincubation).

### 2.6. Griess Assay for Nitrite

Nitrite was measured using the Griess reaction. Fresh aortic vessel segments from each mouse were incubated in phenolred-free media with 10 ng/mL leptin and without. After adding 2% aminobenzenesulphoamide in 2.5% phosphoric acid, vessels were incubated for 5 min in the dark. Thereafter, 0.2% NED-reagent (N-(1-naphthyl) ethylenediamine dihydrochloride) was added and incubated and protected from light for 10 min. Absorbance at 540 nm wavelength was measured in a 96-well plate reader. With the help of a nitrite standard (0–100 µM) and linear regression, the nitrite concentration in samples was calculated. Measurements were performed in triplicate.

### 2.7. Serum Analysis

Serum concentrations of leptin were measured using a Mouse leptin ELISA Kit (Invitrogen, ThermoFisher Scientific, Carlsbad, CA, USA) following the manual’s instructions.

### 2.8. Statistical Analyses

Data are shown as mean ± standard deviation (SD). Statistical analysis was performed by a *t*-test or one-way ANOVA followed by the Holm–Sidak post hoc test or two-way ANOVA, respectively (GraphPad Prism 10; GraphPad Software, Inc., La Jolla, CA, USA). A value of *p* < 0.05 was considered statistically significant. Pearson’s correlational analysis was used and the correlation coefficient (r) was calculated.

## 3. Results

### 3.1. Increased Nox4 Expression under Dyslipidemia Conditions

**Figure 1:**
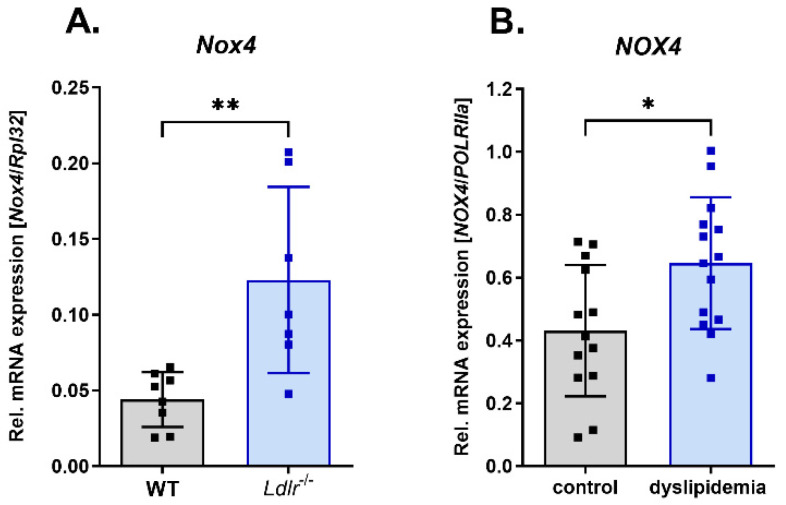
Increased Nox4 expression under dyslipidemia conditions. (**A**) Relative Nox4 mRNA expression in mesenteric artery of WT compared with *Ldlr^−/−^* mice (*n* ≥ 7). (**B**) Relative NOX4 mRNA expression in left internal mammary arteries of patients without and with dyslipidemia undergoing coronary bypass surgery (*n* ≥ 13). Statistics: *t* test; * *p* < 0.05; ** *p* < 0.01.

In the mesenteric arteries of *Ldlr^−/−^* mice, we detected increased *Nox4* mRNA expression compared with wild-type (WT) mice (Figure 1A). In human left internal mammary arteries (LIMA) from patients undergoing coronary bypass surgery, we also found evidence for higher *NOX4* expression in patients with dyslipidemia (Figure 1B).

### 3.2. Nox4 Deletion Led to Endothelial Dysfunction in the Mesenteric Artery

**Figure 2:**
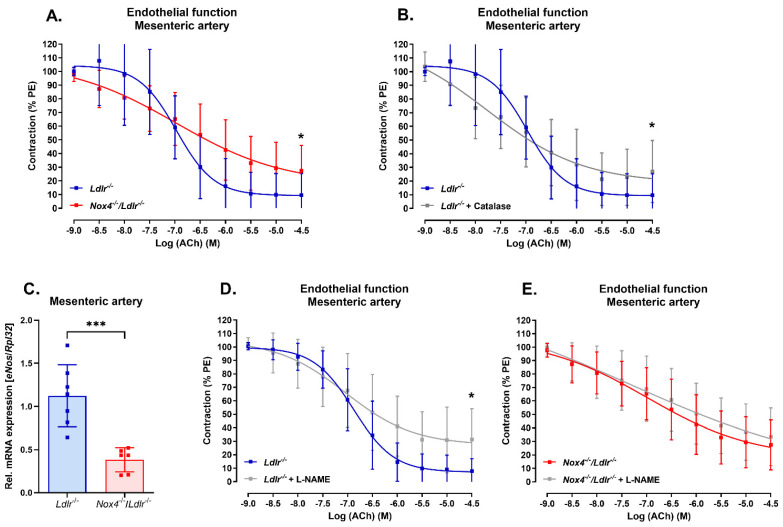
Nox4 deletion led to endothelial dysfunction in mesenteric arteries of *Ldlr^−/−^* mice. *(***A***)* Concentration–response curve for acetylcholine (ACh) in mesenteric arteries of *Ldlr^−/−^* and *Nox4^−/−^/Ldlr^−/−^* mice (*n* ≥ 12). *(***B***)* Concentration–response curve for acetylcholine (ACh) in mesenteric arteries of *Ldlr^−/−^* mice with and without catalase incubation (*n* = 12). *(***C***)* Relative *eNos* mRNA expression in mesenteric arteries of *Ldlr^−/−^* and *Nox4^−/−^/Ldlr^−/−^* mice (*n* ≥ 6). *(***D***)* Concentration–response curve for acetylcholine (ACh) in mesenteric arteries of *Ldlr^−/−^* mice with and without L-NAME incubation (*n* ≥ 6). *(***E***)* Concentration–response curve for acetylcholine (ACh) in mesenteric arteries of *Nox4^−/−^/Ldlr^−/−^* mice with and without L-NAME incubation (*n* ≥ 10). Statistics: (**A**,**B**,**D**,**E**) sigmoidal non-linear curve fit and *t* test of maximal effect; (**C**) *t* test; * *p* < 0.05; *** *p* < 0.001.

To determine the impact of Nox4 in resistance vessels under dyslipidemia conditions, we analyzed the vascular function in mesenteric arteries of *Ldlr^−/−^* mice and Nox4-depleted *Ldlr^−/−^* (*Nox4^−/−^/Ldlr^−/−^*) mice. Endothelial dysfunction was detected in the double knockout *Nox4^−/−^/Ldlr^−/−^* mice (Figure 2A). Similarly, we could induce endothelial dysfunction in the mesenteric arteries of *Ldlr^−/−^* mice when incubated with H_2_O_2_ scavenger catalase, indicating the percentage of H_2_O_2_-mediated vasorelaxation in those resistance arteries (Figure 2B). We previously reported the development of endothelial dysfunction in the aorta of *Nox4^−/−^/Ldlr^−/−^* mice already at an age of 10 weeks. Here, we could also confirm endothelial dysfunction in the aorta of *Nox4^−/−^/Ldlr^−/−^* 26-week-old mice (Supplement Figure S1).

In the mesenteric artery, *eNos* expression was significantly decreased in *Nox4^−/−^/Ldlr^−/−^* mice compared with *Ldlr^−/−^* mice (Figure 2C). Consequently, incubation with nitric oxide synthase inhibitor L-NAME decreased endothelial function only in the mesenteric arteries of *Ldlr^−/−^* mice (Figure 2D), but not in the mesenteric arteries of *Nox4^−/−^/Ldlr^−/−^* mice. This might indicate reduced nitric oxide-mediated vasodilation in the mesenteric arteries when Nox4 is deleted (Figure 2E).

### 3.3. Anti-Contractile Properties of Perivascular Adipose Tissue on Mesenteric Arteries Were Absent in Nox4-Depleted Mice

**Figure 3:**
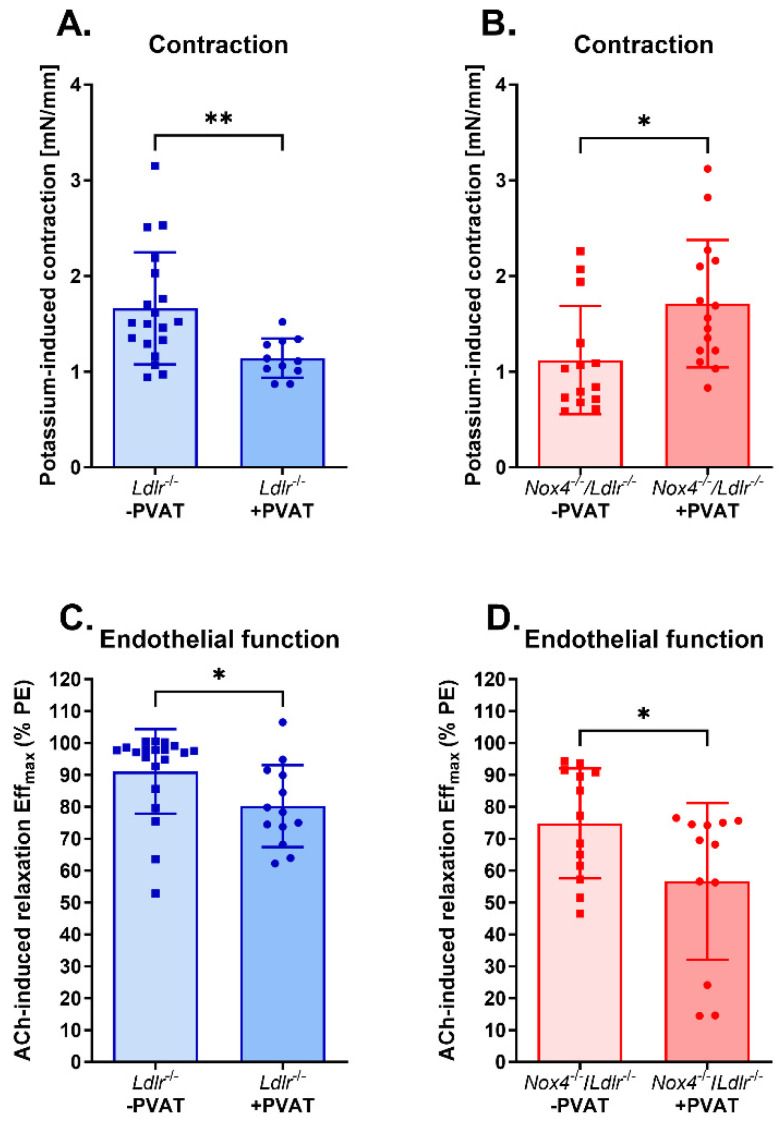
Perivascular adipose tissue reduced contraction only in *Ldlr*^−/−^ mice, but further impaired endothelium-dependent vasodilation in both *Ldlr*^−/−^ and *Nox4*^−/−^/*Ldlr*^−/−^ mice. (**A**) Potassium-induced contraction in mesenteric arteries without (-PVAT) and with perivascular adipose tissue (+PVAT) of *Ldlr*^−/−^ mice (*n* ≥ 12). (**B**) Potassium-induced contraction in mesenteric arteries without (-PVAT) and with perivascular adipose tissue (+PVAT) of *Nox4*^−/−^/*Ldlr*^−/−^ mice (*n* ≥ 9). (**C**) Maximal effect of concentration–response to acetylcholine (ACh) in mesenteric arteries without (-PVAT) and with perivascular adipose tissue (+PVAT) of *Ldlr*^−/−^ mice (*n* ≥ 13). (**D**) Maximal effect of concentration–response to acetylcholine (ACh) in mesenteric arteries without (-PVAT) and with perivascular adipose tissue (+PVAT) of *Nox4*^−/−^/*Ldlr*^−/−^ mice (*n* ≥ 12). Statistics: *t* test; * *p* < 0.05; ** *p* < 0.01.

In the following experiments, we analyzed the vascular function of resistance arteries with surrounding perivascular adipose tissue (PVAT). One of the key attributes of PVAT is the anti-contractile effect on the vessel. We tested that by adding potassium as well as phenylephrine to the vessel segments. Potassium-induced contraction (Figure 3A) and phenylephrine (PE)-induced contraction (Supplement Figure S2A) were significantly reduced with surrounding perivascular adipose tissue (+PVAT) in *Ldlr*^−/−^ mice. In contrast, knockout of *Nox4* caused increased potassium-induced contraction in mesenteric arteries with PVAT (+PVAT) (Figure 3B). The anti-contractile effect of PVAT was also absent in *Nox4*^−/−^/*Ldlr*^−/−^ mice when tested with phenylephrine (Supplement Figure S2B).

Next, we analyzed endothelium-dependent vasorelaxation in the presence of PVAT. The maximal effect of acetylcholine on vasorelaxation was significantly diminished by perivascular adipose tissue (+PVAT) in *Ldlr*^−/−^ mice (Figure 3C). Mesenteric arteries with perivascular adipose tissue (+PVAT) from *Nox4*^−/−^/*Ldlr*^−/−^ mice showed even further decreased maximal endothelium-dependent vasorelaxation (Figure 3D).

### 3.4. Expression of Adipokines in Perivascular Adipose Tissue from Mesenteric Arteries of Nox4^−/−^/Ldlr^−/−^ Mice and Ldlr^−/−^ Mice and Comparison to Perivascular Adipose Tissue from Aortas

**Figure 4:**
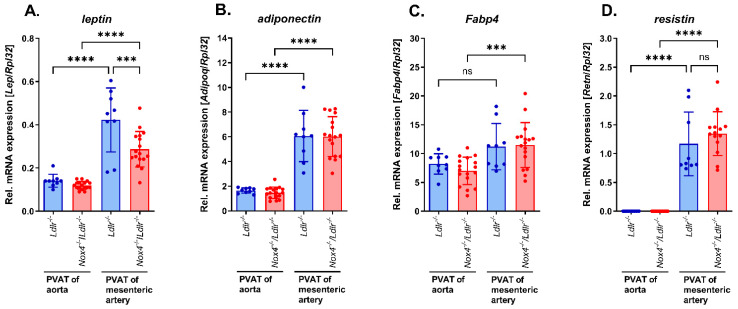
Expression of adipokines in perivascular adipose tissue (PVAT) from aorta and mesenteric artery of *Nox4*^−/−^/*Ldlr*^−/−^ and *Ldlr*^−/−^ mice. (**A**) Relative leptin (*Lep*) mRNA expression, (**B**) relative adiponectin (*Adipoq*) mRNA expression, (**C**) relative fatty acid binding protein 4 (*Fabp4*) mRNA expression and (**D**) relative resistin (*Retn*) expression in PVAT from aorta and mesenteric artery of *Ldlr*^−/−^ and *Nox4*^−/−^/*Ldlr*^−/−^ mice (*n* ≥ 9). Statistics: two-way ANOVA; *** *p* < 0.001; **** *p* < 0.0001; ns, not significant.

To gain a better understanding of the functional differences, we conducted comparison analysis of PVAT from mesenteric arteries of *Ldlr*^−/−^ and *Nox4*^−/−^/*Ldlr*^−/−^ mice. Furthermore, to evaluate the expression pattern of white and brown/beige adipocyte markers, we included PVAT from the aortas of *Ldlr*^−/−^ and *Nox4*^−/−^/*Ldlr*^−/−^ mice in the comparison. Expression of markers for white adipocytes significantly differed between both types of PVAT. In both mouse strains, leptin (*Lep*) mRNA expression was significantly higher in PVAT from mesenteric arteries compared to thoracic aorta (Figure 4A). However, PVAT from *Nox4*^−/−^/*Ldlr*^−/−^ mice expressed significantly less leptin than PVAT from mesenteric arteries of *Ldlr*^−/−^ mice without differences in serum leptin levels and body weight between both mouse strains (Figure 4A and Supplement Figures S3 and S4). Expression of adiponectin (*Adipoq*) (Figure 4B), fatty acid binding protein 4 (*Fabp4*) (Figure 4C) and resistin (*Retn*) (Figure 4D) showed a similarly higher expression in PVAT from mesenteric arteries compared with PVAT from the aorta, independent of the genotype.

### 3.5. Marker of Brown/Beige Adipocytes in Aortic and Mesenteric Perivascular Adipose Tissue from Nox4^−/−^/Ldlr^−/−^ Mice Compared to Ldlr^−/−^ Mice

**Figure 5:**
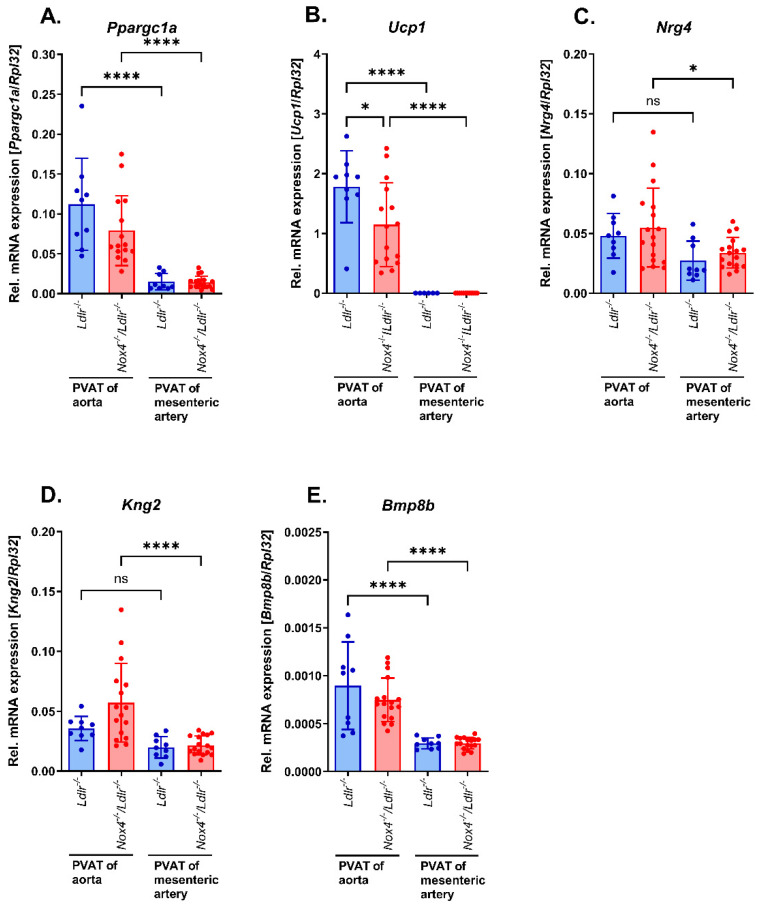
Marker expression of brown/beige adipocytes in perivascular adipose tissue (PVAT) from aorta and mesenteric arteries of *Nox4*^−/−^/*Ldlr*^−/−^ and *Ldlr*^−/−^ mice. (**A**) Relative Pparg coactivator 1 alpha (*Ppargc1a*) mRNA expression, (**B**) relative uncoupling protein 1 (*Ucp1*) mRNA expression, (**C**) relative neuregulin 4 (*Nrg4*) mRNA expression, (**D**) relative bone morphogenetic protein 8b (*Bmp8b*) mRNA expression and (**E**) relative kininogen 2 (*Kng2*) mRNA expression in PVAT from aorta and mesenteric arteries of *Ldlr*^−/−^ and *Nox4*^−/−^/*Ldlr*^−/−^ mice (*n* ≥ 9). Statistics: two-way ANOVA; * *p* < 0.05; **** *p* < 0.0001; ns, not significant.

Expression of primary indicators for brown and beige adipocytes revealed prominent expression in PVAT from the thoracic aorta and rather weak expression in PVAT from mesenteric arteries. Peroxisome proliferative-activated receptor gamma, coactivator 1 alpha (*Ppargc1a*) (Figure 5A) and mitochondrial brown fat uncoupling protein 1 (*Ucp1*) (Figure 5B) were similarly significantly elevated in murine PVAT from thoracic aortas compared with PVAT from mesenteric arteries. However, only *Ucp1* was less expressed in aortic PVAT from *Nox4*^−/−^/*Ldlr*^−/−^ compared with *Ldlr*^−/−^ mice. In addition, brown and beige adipocyte markers bone morphogenetic protein 8b (*Bmp8b*) (Figure 5C), neuregulin 4 (*Nrg4*) (Figure 5D) and kininogen 2 (*Kng2*) (Figure 5E) were analyzed. We observed a similar pattern of brown/beige markers being significantly higher expressed in aortic PVAT compared with mesenteric artery PVAT. Nevertheless, we could not detect significant differences in the expression of these genes between *Ldlr*^−/−^ and *Nox4*^−/−^/*Ldlr*^−/−^ mice.

### 3.6. Adipokine Profile in Human Perivascular Adipose Tissue: Correlation of Leptin and NOX4 Expression in Human Perivascular Adipose Tissue

**Figure 6:**
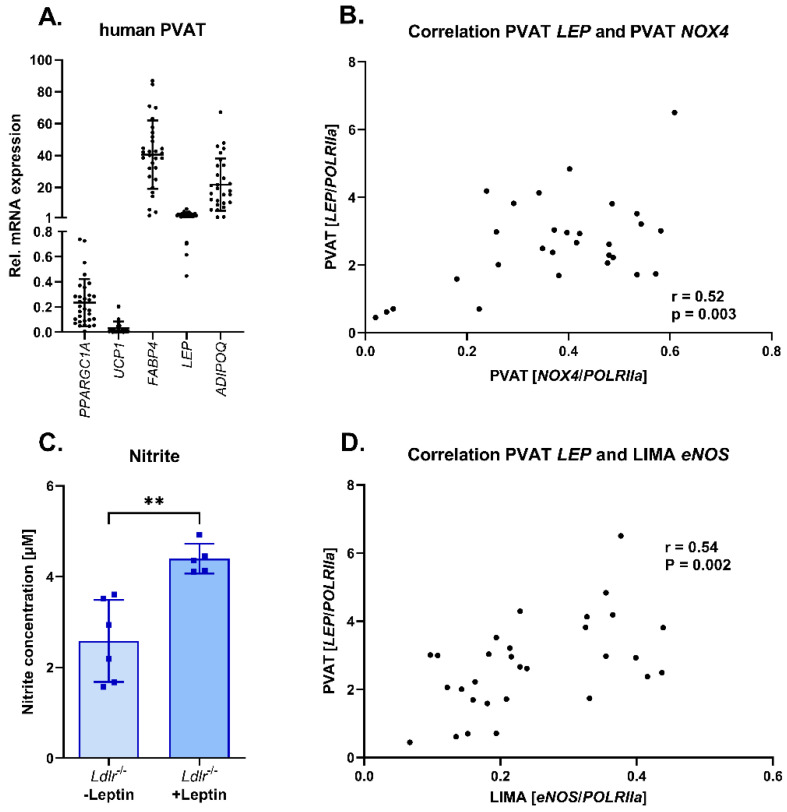
Correlation between *NOX4* and *LEP* mRNA expression in human perivascular adipose tissue (PVAT). (**A**) Relative *PPARGC1A*, *UCP1*, *FABP4*, *LEP* and *ADIPOQ* expression in human perivascular adipose tissue from left internal mammary arteries (LIMA). (**B**) Correlation of *NOX4* and *LEP* mRNA expression in perivascular adipose tissue from left internal mammary arteries (*n* = 29). (**C**) Nitrite concentration in vessel segments from *Ldlr*^−/−^ mice with and without leptin treatment (*n* ≥ 5). (**D**) Correlation of *LEP* mRNA expression in perivascular adipose tissue from left internal mammary arteries and *eNOS* expression in left internal mammary arteries (*n* = 30). Statistics: (**C**) *t* test; ** *p* < 0.01 (**B**,**D**) Pearson’s correlation coefficient (r).

We were able to show severe differences in the expression of adipocyte markers with higher white adipocyte marker expression in PVAT from murine mesenteric arteries. Additionally, in those resistance arteries, we had observed that deletion of Nox4 resulted in changes in leptin expression in PVAT. To extend these findings from murine models to humans, we analyzed PVAT from human left internal mammary arteries. Here, we found a higher expression profile for white adipocyte markers including fatty acid binding protein 4 (FABP4), leptin (LEP) and adiponectin (ADIPOQ) and lower expression of brown adipocyte markers peroxisome proliferative-activated receptor gamma, coactivator 1 alpha (PPARGC1A) and uncoupling protein 1 (UCP1) (Figure 6A). Analysis of NOX4 mRNA expression and leptin (LEP) mRNA expression in human PVAT revealed a correlation, indicating a possible link between both also in humans (Figure 6B). To test the potential of leptin released by PVAT to induce eNOS activation, we exposed vessel segments to leptin in vitro and quantified nitrite concentrations in the supernatant. We detected increased nitrite concentrations as an indicator of increased nitric oxide synthase activity (Figure 6C). Finally, we were able to correlate leptin expression in the human PVAT from left internal mammary arteries with *eNOS* expression in the left internal arteries (Figure 6D).

## 4. Discussion

In the present study, we provide evidence that *Nox4* is important for the maintenance of vascular function in resistance arteries under conditions of dyslipidemia. We observed increased *NOX4* expression in arterial vessels from mice and humans under dyslipidemia. For human macrophages, it has already been reported that oxidized low-density lipoprotein (oxLDL) induced *NOX4* expression [28]. Likewise, it has been reported that endothelial progenitor cells from hyperlipidemic patients exhibited increased *NOX4* expression and activity [29]. Hypercholesterolemia is also known to induce TGF-β signaling, which in turn induces *NOX4* [30,31].

On a functional level, we observed that a loss of *Nox4* led to an altered endothelial function in small resistance arteries. Previously, we had reported that already in young hypercholesterolemic mice, depletion of *Nox4* caused endothelial dysfunction of the aorta which further progressed after high-fat diet feeding to a higher plaque burden in the aorta [25]. Ray et al. found improved endothelium-dependent relaxation in the aorta and coronary artery of mice with endothelium-specific Nox4 overexpression [22]. It had also been shown that endothelial cells depleted of *Nox4* had reduced *eNos* expression, *Nrf-2* expression and subsequent heme oxygenase-1 expression [23]. In the mesenteric arteries of double knockout *Nox4^−/−^/Ldlr^−/−^* mice, we observed significantly reduced *eNos* expression as well. Therefore, inhibiting nitric oxide synthase in the mesenteric arteries of *Nox4*^−/−^/*Ldlr*^−/−^ mice did not further impair endothelial dysfunction.

Perivascular adipose tissue (PVAT) exhibits anti-contractile properties, which we also observed in *Ldlr*^−/−^ mice. However, in mice lacking *Nox4*, PVAT surrounding mesenteric arteries did not further decrease contractility. Additionally, PVAT on mesenteric arteries aggravated endothelial dysfunction in both *Ldlr*^−/−^ and *Nox4*^−/−^/*Ldlr*^−/−^ mice. Gao et al. showed that PVAT of the aorta decreased vascular contraction by releasing a factor that induced endothelium-dependent relaxation [7]. Among those factors discussed to mediate the anticontractile properties of PVAT is leptin [32]. Decreased leptin expression in perivascular adipose tissue from *Nox4*^−/−^/*Ldlr*^−/−^ mice might account for the loss of anticontractile effects observed in PVAT of the mesenteric arteries in these mice. In spontaneously hypertensive rats with decreased leptin expression in their PVAT, the anticontractile effect and endothelium-dependent vasorelaxation were reduced [32]. The vasodilatory effect of leptin has also been shown in human vessels [33]. Leptin treatment of vessels from *Ldlr*^−/−^ mice led to an increase in nitrite production, indicating activation of nitric oxide synthase. Leptin was reported to activate nitric oxide synthase through AKT and JAK2 [34]. However, in obesity, the mechanism might be different. Here, hyperleptinemia rather promoted eNOS uncoupling and endothelial dysfunction [35,36].

Experimental studies have shown that inhibition of NOX enzymes in adipocytes can affect leptin secretion and adipocyte differentiation [37]. Preadipocytes treated with siRNA against NOX4 exhibited fewer accumulated fat droplets. NOX4 promoted adipocyte differentiation via MAP kinase phosphatase-1 [38]. Additionally, NOX-derived reactive oxygen species have been implicated in leptin-induced effects [39,40].

In our study, comparison of the adipokine profile revealed an increased white adipocyte pattern and decreased brown/beige adipocyte marker expression in the PVAT of mesenteric arteries. In contrast, PVAT from the aorta showed increased expression of brown/beige marker genes. Mestres-Arenas et al. also observed batokines such as uncoupling protein-1 (*Ucp1*), peroxisome proliferator-activated receptor gamma coactivator 1-alpha (*Ppargc1a*), neuregulin 4 (*Nrg4*), kininogen-2 (*Kng2*) and bone morphogenetic protein-8b (*Bmp8b*) in murine thoracic PVAT that can even be increased by cold exposure [41].

It has been reported that leptin might stimulate β3-adrenergic receptors leading to upregulation of *Ucp1* [42]. We were able to detect differences in *Ucp1* expression in the more brown/beige thoracic aortic PVAT.

In human PVAT from internal mammary arteries, we observed that *NOX4* expression correlated with leptin expression. Comparison of PVAT from internal mammary arteries and PVAT from coronary arteries indicated reduced leptin, IL-1β and IL-6 expression in PVAT from internal mammary arteries [43]. Thus, analyzing the effects of NOX4 in rather inflammatory PVAT might shed further light into its role in dyslipidemia, obesity, endothelial dysfunction and hypertension [14,15,44].

## 5. Conclusions

We provide evidence that NOX4 is essential for the vascular function of resistance arteries, particularly under dyslipidemia conditions. Deletion of *Nox4* led to decreased *eNOS* expression and impaired nitric oxide-mediated relaxation in mesenteric arteries. We showed that PVAT under these conditions further impaired vascular function. The adipokine profile revealed decreased leptin expression and further decreased eNOS expression and activity.

## 6. Limitations

The focus of our investigation was the examination of resistance arteries and the perivascular adipose tissue enveloping them. Unfortunately, due to constraints in accessing human resistance arteries with perivascular adipose tissue, our analysis was restricted to internal mammary arteries with perivascular adipose tissue, thereby representing a limitation of our study. Additionally, our objectives were the investigation of Nox4-derived H_2_O_2_-mediated vasodilation and NO-mediated vasodilation specifically within resistance arteries. Nonetheless, the mechanisms underlying vasorelaxation in mesenteric arteries encompass additional factors such as prostaglandins (PGs), prostacyclin and other endothelium-derived hyperpolarizing factors (EDHFs), which, while not directly explored in this study, may differ between *Ldlr*^−/−^ and *Nox4*^−/−^/*Ldlr*^−/−^ mice.

## Figures and Tables

**Table 1:** Patient data: control group vs. patients with dyslipidemia. Data are shown as mean ± SD. Abbreviations: ACE, angiotensin-converting enzyme; ARB, angiotensin receptor blocker; BMI, body mass index.

	Control Group	Patients with Dyslipidemia	*p*-Value
Number of patients	14	15	
Dyslipidemia [yes = 1; no = 0]	0	1	
Age [years]	70.07 ± 8.02	71.00 ± 10.13	0.79
Sex [% male]	100	100	
BMI [kg/m^2^]	28.75 ± 3.74	29.60 ± 3.28	0.57
Smoking [yes = 1; no = 0]	0.43 ± 0.51	0.47 ± 0.52	0.84
Diabetes mellitus type 2 [yes = 1; no = 0]	0.71 ± 0.47	0.67 ± 0.49	0.79
Hypertension [yes = 1; no = 0]	0.93 ± 0.27	0.93 ± 0.26	0.96
Ejection fraction [%]	39.67 ± 17.08	39.63 ± 13.10	0.99
ACE inhibitor [yes = 1; no = 0]	0.79 ± 0.43	0.60 ± 0.51	0.30
Beta blocker [yes = 1; no = 0]	0.86 ± 0.36	0.93 ± 0.26	0.52
Statin [yes = 1; no = 0]	0.86 ± 0.36	0.87 ± 0.35	0.94
Aspirin [yes = 1; no = 0]	0.86 ± 0.36	0.87 ± 0.35	0.94
Diuretics [yes = 1; no = 0]	0.57 ± 0.51	0.73 ± 0.46	0.38
ARB [yes = 1; no = 0]	0.14 ± 0.36	0.20 ± 0.41	0.70

**Table 2:** Primers used for real-time PCR.

Gene	Primers	Sequence, 5′-3′	Accession Number
*Human ADIPOQ*	Forward	TCCTCACTTCCATTCTGACTGC	NM_001177800.1
	Reverse	GTAGAACAGCTCCCAGCAACA	
*Murine Adipoq*	Forward	CAGTGGATCTGACGACACCAA	NM_009605.5
	Reverse	ACGTCATCTTCGGCATGACTG	
*Murine Bmp8b*	Forward	TCCGCCTATTACTGTGCTGG	NM_007559.5
	Reverse	TAGGCACACAGCACACCTTG	
*Human eNOS*	Forward	GAACCTGTGTGACCCTCACC	NM_000603.5, NM_001160109.2, NM_001160110.1, NM_001160111.1
	Reverse	TGGCTAGCTGGTAACTGTGC	
*Murine eNos*	Forward	CTCATGGGCACGGTGATG	NM_008713.4
	Reverse	ACCACATCATACTCATCCAT	
*Human FABP4*	Forward	GAAAACTGCAGCTTCCTTCTCAC	NM_001442.3
	Reverse	CTGGTGGCAAAGCCCACTC	
*Murine Fabp4*	Forward	TGGGAACCTGGAAGCTTGTC	NM_001409513.1, NM_001409514.1, NM_024406.4
	Reverse	CTTTCCTTGTGGCAAAGCCC	
*Murine Kng2*	Forward	CGACTGCAATGCTAACGTGT	NM_001102409.1, NM_001102410.1, NM_201375.2
	Reverse	AGGCCTCCTTCGGATAGGAAT	
*Human LEP*	Forward	CAAGCTGTGCCCATCCAAAAA	NM_000230.2
	Reverse	TGAAGTCCAAACCGGTGACT	
*Murine Lep*	Forward	TGCTGCAGATAGCCAATGAC	NM_008493.3
	Reverse	GAGTAGAGTGAGGCTTCCAGGA	
*Human NOX4*	Forward	TAACCTCAACTGCAGCCTTATC	NM_001143836.3, NM_001143837.2, NM_001291926.2, NM_001291927.1, NM_001291929.2, NM_001300995.1, NM_016931.5
	Reverse	CTTTTATCCAACAATCTCCTGGTTCTC	
*Murine Nox4*	Forward	TGTTGGGCCTAGGATTGTGTT	NM_001285833.1, NM_001285835.1, NM_015760.5
	Reverse	AGGGACCTTCTGTGATCCTCG	
*Murine Nrg4*	Forward	CCTACTATCCCCAGCCCATTCT	NM_032002.3, NM_001425100.1
	Reverse	TGCCGACAGATTACTTTCGCT	
*Human PPARGC1a*	Forward	CTTTGCGCAGGTCAAACGAA	NM_001330753.1, NM_001330752.1, NM_013261.4, NM_001330751.1
	Reverse	GGTGGAAGCAGGGTCAAAGT	
*Murine Ppargc1a*	Forward	AATGCAGCGGTCTTAGCACT	NM_008904.2
	Reverse	TCTCGGTCTTAACAATGGCAGG	
*Human POLRIIa*	Forward	ACCTGCGGTCCACGTTGTGT	NM_000937.4
	Reverse	CCACCATTTCCCCGGGATGCG	
*Murine Retn*	Forward	TGTCCCATCGATGAAGCCAT	NM_001204959.1
	Reverse	TGGAGGAGACTGTCCAGCAA	
*Murine Rpl32*	Forward	GCGCTGCCTACGAGGTGGCTG	NM_172086.2
	Reverse	CTGGCCCTTGAACCTTCTCCGC	
*Human UCP1*	Forward	CTAACGAAGGACCAACGGCT	NM_021833.5
	Reverse	ACGTTCCAGGATCCAAGTCG	
*Murine Ucp1*	Forward	TACCCAAGCGTACCAAGCTG	NM_009463.3
	Reverse	ACCCGAGTCGCAGAAAAGAA	

## Data Availability

The raw data supporting the conclusions of this article will be made available by the authors on request.

## Supplementary Materials

The following supporting information can be downloaded at: https://www.mdpi.com/article/10.3390/antiox13050503/s1, Supplement Figure S1: Endothelium-dependent vasorelaxation in the aorta of 26-week-old *Ldlr*^−/−^ and *Nox4*^−/−^/*Ldlr*^−/−^ mice; Supplement Figure S2: Phenylephrine-induced contraction in mesenteric arteries without and with perivascular adipose tissue of *Ldlr*^−/−^ and *Nox4*^−/−^/*Ldlr*^−/−^ mice; Supplement Figure S3: Leptin serum concentration in *Ldlr*^−/−^ and *Nox4*^−/−^/*Ldlr*^−/−^ mice; Supplement Figure S4: Body weight of *Ldlr*^−/−^ and *Nox4*^−/−^/*Ldlr*^−/−^ mice.
