# Supplementary material for: The Vascular Function of Resistance Arteries Depends on NADPH Oxidase 4 and Is Exacerbated by Perivascular Adipose Tissue
Source: Antioxidants (Basel). 2024 Apr 23;13(5):503. doi: 10.3390/antiox13050503 (PMC11118120; doi:10.3390/antiox13050503)
Supplement: Supplementary file 1 [file antioxidants-13-00503-s001.zip › antioxidants-2949598-supplementary.pdf]

Supplementary Materials

The Vascular Function of Resistance Arteries Depends on NADPH Oxidase 4 and Is Exacerbated by Perivascular Adipose Tissue

Patrick Diaba-Nuhoho †, Jennifer Mittag †, Coy Brunssen, Henning Morawietz * and Heike Brendel *

Division of Vascular Endothelium and Microcirculation, Department of Medicine III, University Hospital and Faculty of Medicine Carl Gustav Carus, TUD Dresden University of Technology, Fetscherstr. 74, 01307 Dresden, Germany

* Correspondence: henning.morawietz@tu-dresden.de (H.M.); heike.brendel@uniklinikum-dresden.de (H.B.); Tel.: +49-351-4586625; (H.M.); +49-351-45816678 (H.B.); Fax: +49-351-4586354 (H.M.)

† These authors contributed equally to this study.

Supplement Figures

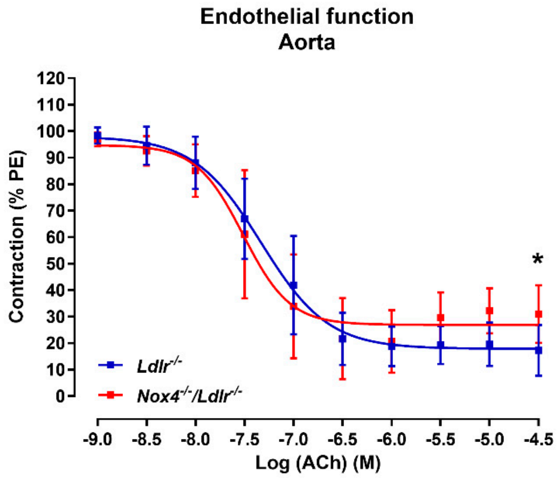

Supplement Figure S1. Endothelium-dependent vasorelaxation in the aorta of 26-week-old *Ldlr*$^{-/-}$ and *Nox4*$^{-/-}$/*Ldlr*$^{-/-}$ mice.

Concentration-response curve for acetylcholine (ACh) in aortic segments of *Ldlr*$^{-/-}$ and *Nox4*$^{-/-}$/*Ldlr*$^{-/-}$ mice ($n \geq 9$). Statistics: repeated measures ANOVA; *$p < 0.05$.

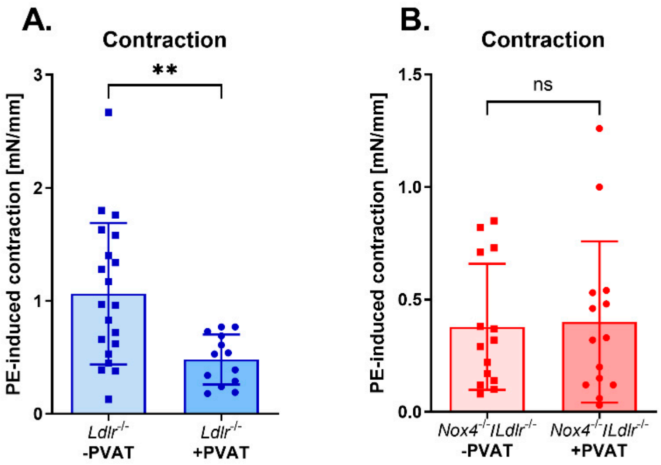

Supplement Figure S2. Phenylephrine-induced contraction in mesenteric arteries without and with perivascular adipose tissue of *Ldlr*$^{-/-}$ and *Nox4*$^{-/-}$/*Ldlr*$^{-/-}$ mice.

(A) Phenylephrine (PE)-induced contraction in mesenteric arteries without (-PVAT) and with perivascular adipose tissue (+PVAT) of *Ldlr*$^{-/-}$ mice ($n \geq 13$). **(B)** Phenylephrine (PE)-induced contraction in mesenteric arteries without (-PVAT) and with perivascular adipose tissue (+PVAT) of *Nox4*$^{-/-}$/*Ldlr*$^{-/-}$ mice ($n \geq 14$). Statistics: t test; ** $p < 0.01$, ns, not significant.

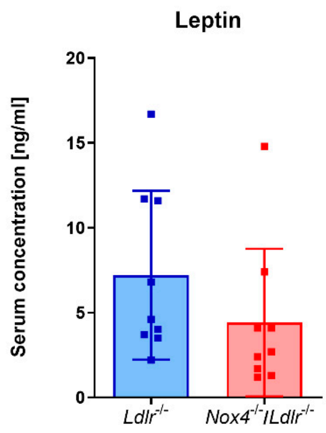

Supplement Figure S3. Leptin serum concentration in *Ldlr*$^{-/-}$ and *Nox4*$^{-/-}$/*Ldlr*$^{-/-}$ mice. Leptin serum concentration in 26-week-old *Ldlr*$^{-/-}$ and *Nox4*$^{-/-}$/*Ldlr*$^{-/-}$ mice. ($n \geq 9$).

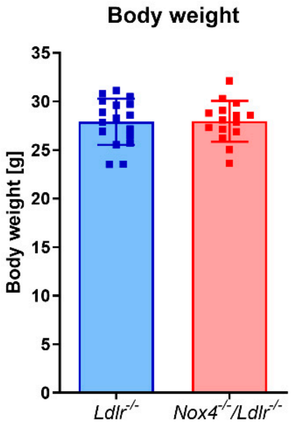

Supplement Figure S4. Body weight of *Ldlr*$^{-/-}$ and *Nox4*$^{-/-}$/*Ldlr*$^{-/-}$ mice.
Body weight of 26-week-old *Ldlr*$^{-/-}$ and *Nox4*$^{-/-}$/*Ldlr*$^{-/-}$ mice ($n \geq 15$).
